# D-pinitol Inhibits Prostate Cancer Metastasis through Inhibition of αVβ3 Integrin by Modulating FAK, c-Src and NF-κB Pathways

**DOI:** 10.3390/ijms14059790

**Published:** 2013-05-08

**Authors:** Tien-Huang Lin, Tzu-Wei Tan, Tsung-Hsun Tsai, Chi-Cheng Chen, Teng-Fu Hsieh, Shang-Sen Lee, Hsin-Ho Liu, Wen-Chi Chen, Chih-Hsin Tang

**Affiliations:** 1 School of Chinese Medicine, China Medical University, Taichung 404, Taiwan; E-Mail: thlin@hotmail.com; 2 Department of Urology, Buddhist Tzu Chi General Hospital Taichung Branch, Taichung 427, Taiwan; E-Mails: d90443001@ntu.edu.tw (T.-H.T.); kukoc0925@gmail.com (C.-C.C.); hdf95@yahoo.com.tw (T.-F.H.); j520037@yahoo.com.tw (S.-S.L.); uroliu@gmail.com (H.-H.L.); 3 Graduate Institute of Basic Medical Science, China Medical University, Taichung 404, Taiwan; E-Mail: twtan@mail.cmu.edu.tw; 4 Department of Pharmacology, School of Medicine, China Medical University, Taichung 404, Taiwan; 5 Graduate Institute of Integrated Medicine, China Medical University, Taichung 404, Taiwan; 6 Department of Urology, China Medical University Hospital, Taichung 404, Taiwan; 7 Department of Biotechnology, College of Health Science, Asia University, Taichung 413, Taiwan

**Keywords:** D-pinitol, integrin, prostate cancer, migration, FAK

## Abstract

Prostate cancer is the most commonly diagnosed malignancy in men and shows a predilection for metastasis to the bone. D-pinitol, a 3-methoxy analogue of d-chiro-inositol, was identified as an active principle in soy foods and legumes, and it has been proven to induce tumor apoptosis and metastasis of cancer cells. In this study, we investigated the anti-metastasis effects of D-pinitol in human prostate cancer cells. We found that D-pinitol reduced the migration and the invasion of prostate cancer cells (PC3 and DU145) at noncytotoxic concentrations. Integrins are the major adhesive molecules in mammalian cells and have been associated with the metastasis of cancer cells. Treatment of prostate cancer cells with D-pinitol reduced mRNA and cell surface expression of αvβ3 integrin. In addition, D-pinitol exerted its inhibitory effects by reducing focal adhesion kinase (FAK) phosphorylation, c-Src kinase activity and NF-κB activation. Thus, D-pinitol may be a novel anti-metastasis agent for the treatment of prostate cancer metastasis.

## 1. Introduction

Prostate cancer is the most commonly diagnosed malignancy in the U.S. and other Western countries [1]. Surgery is the most frequent therapeutic intervention in the early stages of prostate cancer. In advanced stages of prostate cancer, more systemic interventions are required to inhibit the growth and spread of secondary metastases. Bone is a common site of prostate cancer metastasis, which often causes acute pain and bone fractures. Bone metastasis has prognostic value in prostate cancer, because the extent of disease in the bone significantly affects survival [2].

Cancer metastasis is a critical step in tumor progression and the major cause of mortality for patients with cancer. Cell adhesion molecules, such as integrin, cadherin and immunoglobulin superfamilies, have been studied extensively in the context of tumor progression and metastasis [3]. Integrins are a family of transmembrane glycoprotein adhesion receptors that play central roles in the biology of metazoans by controlling cell adhesion, migration, differentiation and apoptosis. Integrins form heterodimers of α and β subunits [4]. There are at least 19 α subunits and eight β subunits that can associate to form 25 unique integrin heterodimers [5,6]. It has been reported that integrins play a central role for many extracellular matrix (ECM) proteins, such as collagen, fibronectin, laminin, osteopontin and vitronectin [7]. In addition, integrins have also been implicated in the metastasis of lung, chondrosarcoma and prostate cancers [8–10]. On the other hand, αvβ3 integrin has been implicated in prostate cancer progression with effects on angiogenesis, survival and invasion [11,12]. An *in vitro* study has found that integrins facilitated prostate cancer cell adhesion, migration through several ECM substrates and transendothelial migration [11].

D-pinitol, a 3-methoxy analogue of D-chiro-inositol, was identified as an active principle in soy foods and legumes [13]. Mature and dried soybean seeds contain up to 1% D-pinitol. D-pinitol functions in plants as an osmolyte by improving the tolerance to drought stress or heat stress. D-pinitol reduces the negative effects of osmotic stress and increases the tolerance of plant tissues to water deficiencies. In addition, D-pinitol possess multifunctional properties, including feeding stimulant, anti-inflammatory, cardioprotective, anti-hyperlipidemic and creatine retention promotion properties [14–16]. Recent studies have shown potential chemotherapeutic efficacy against cancers of the lung, bladder and breast [17,18]. In addition, D-pinitol has been reported to reduce metastasis of human lung cancers [19]. However, the effects of D-pinitol in metastasis of human prostate cancer cells are largely unknown. Here, we report that D-pinitol inhibits the migration and the invasion of human prostate cancer cells. In addition, downregulation of αvβ3 integrin through focal adhesion kinase (FAK), c-Src and NF-κB are involved in D-pinitol-reduced cell motility. Therefore, our data provide evidence that D-pinitol may be an anti-metastatic agent for the treatment of prostate cancer metastasis.

## 2. Results

### 2.1. D-pinitol Does Not Induce Cell Death in Human Prostate Cancer Cells

**Figure 1:**
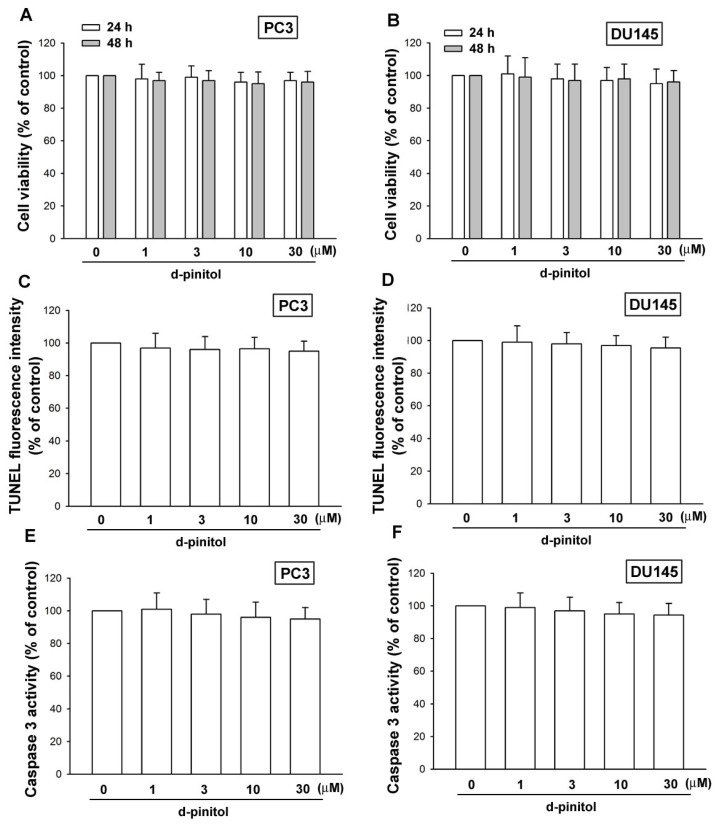
D-pinitol does not induce cell apoptosis in human prostate cancer cells. (**A** and **B**) Cells were incubated with various concentrations of D-pinitol for 24 or 48 h, and the cell viability was examined by MTT assay (*n* = 5). (**C** and **D**) Cells were incubated with D-pinitol for 24 h; the TUNEL positive cells were examined by flow cytometry (*n* = 5). (**E** and **F**) Cells were incubated with D-pinitol for 24 h, and the caspase 3 activity was examined by a caspase 3 ELISA kit (*n* = 4). Results are expressed as the mean ± SE.

D-pinitol has been reported to induce cell death in human cancer cells [17,18]. The cytotoxic effect of D-pinitol in human prostate cancer cells was examined by the MTT assay. Treatment of prostate cancer cells (PC3 and DU145) for 24 or 48 h did not affect cell viability (Figure 1A,B). We next investigated whether D-pinitol induced cell apoptosis in human prostate cancers by TUNEL staining and caspase 3 activity assay. Incubation of the cells with D-pinitol did not enhance TUNEL expression or caspase 3 activity (Figure 1C–F). These data indicate that D-pinitol does not induce cell death in prostate cancer cells. Thus, all subsequent experiments used this D-pinitol concentration range.

### 2.2. D-pinitol Reduces Cell Migration, Wound-Healing Migration and Invasion of Human Prostate Cancer Cells

**Figure 2:**
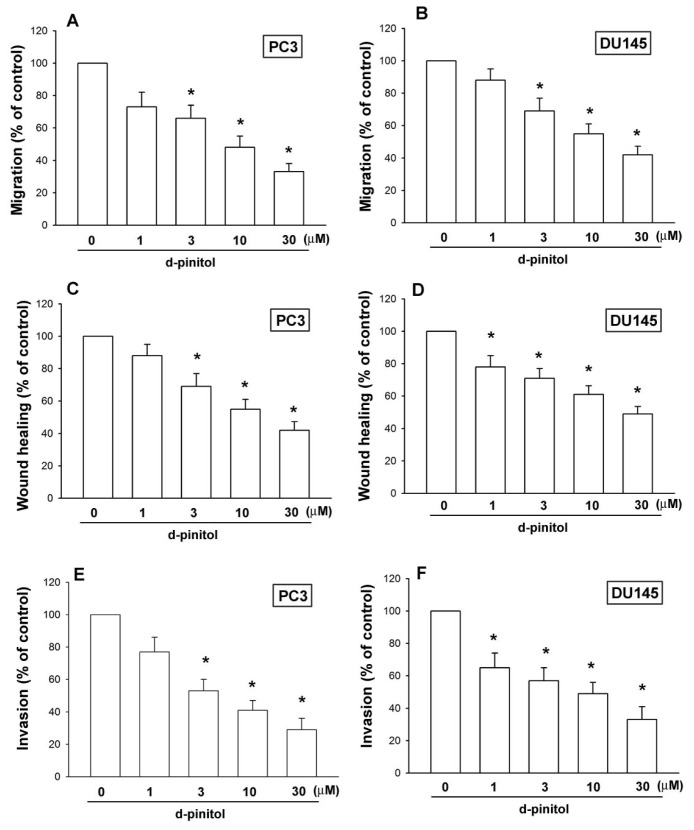
D-pinitol inhibits the migration and the invasion of human prostate cancer cells. (**A**–**F**) Cells were incubated with various concentrations of D-pinitol for 24 h; the cell migration and invasion was examined by Transwell, wound healing and invasion assay (*n* = 4–5). Results are expressed as the mean ± SE. *p < 0.05 compared with control.

D-pinitol has been reported to reduce metastasis of human lung cancers [19]. Therefore, we examined whether D-pinitol inhibits the cell motility of prostate cancer cells. Stimulation of prostate cancer cells with D-pinitol (1–30 μM) dramatically decreased migration in both prostate cancer cell lines (Figure 2A,B). In addition, the wound-scratching assay demonstrated that D-pinitol reduced wound healing activity in prostate cancer cells (Figure 2C,D). We also observed that D-pinitol reduced the invasive ability of prostate cancer cells through a Matrigel basement membrane matrix (Figure 2E,F). Therefore, D-pinitol reduces cell migration and invasion of human prostate cancer cells.

### 2.3. Involvement of αvβ3 Integrin Downregulation in D-pinitol-Reduced Cell Migration of Prostate Cancer Cells

**Figure 3:**
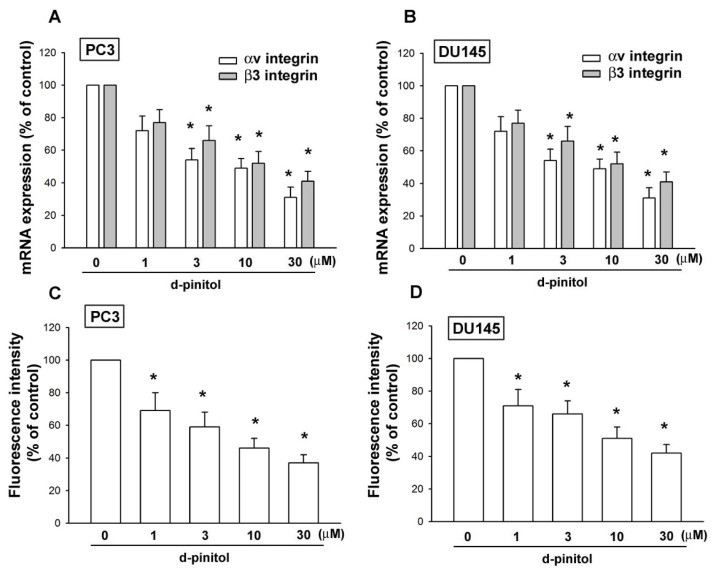
D-pinitol inhibits αvβ3 integrin expression in prostate cancer cells. (**A**–**D**) Cells were incubated with various concentrations of D-pinitol for 24 h; the mRNA and cell surface expression of αvβ3 integrin was examined by qPCR and flow cytometry (*n* = 4–5). Results are expressed as the mean ± SE. *p < 0.05 compared with control.

A previous study has shown αvβ3 integrin activation mediates the migration and the metastasis of human prostate cancer cells [20]. Therefore, we hypothesized that αvβ3 integrin may be involved in the D-pinitol-decreased migration of human prostate cancer cells. Treatment of prostate cancer cells with D-pinitol reduced the mRNA expression of αv and β3 integrin in a concentration-dependent manner (Figure 3A,B). In addition, D-pinitol also reduced the cell surface expression of αvβ3 integrin (Figure 3C,D). Therefore, the downregulation of αvβ3 integrin is involved in the D-pinitol-inhibited migration of prostate cancer cells.

### 2.4. The Effect of D-pinitol on FAK and c-Src Signaling Pathways

**Figure 4:**
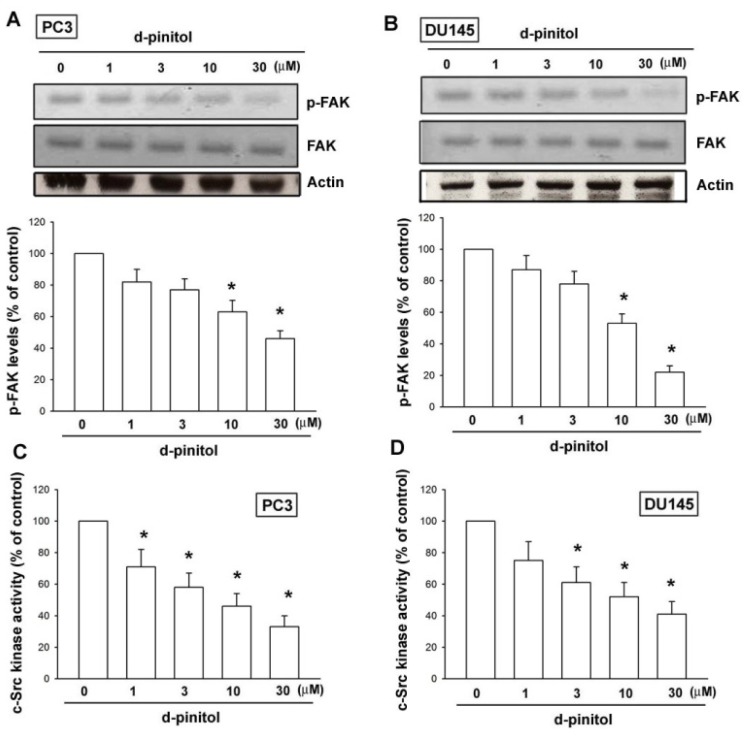
FAK and c-Src pathways affect the D-pinitol response in human prostate cancer cells. (**A** and **B**) Cells were incubated with various concentrations of D-pinitol for 24 h; the p-FAK expression was examined by Western blotting (*n* = 4). (**C** and **D**) Cells were incubated with various concentrations of D-pinitol for 24 h; the c-Src kinase activity was examined by c-Src kinase activity kit (*n* = 5). Results are expressed as the mean ± SE. *p < 0.05 compared with control.

It is well established that FAK-dependent c-Src activation is involved in tumor migration and invasion [21,22]. After the inhibitory effect of D-pinitol on the cell migration and integrin expression was revealed, the effects of D-pinitol on the expression of the FAK and c-Src pathway were investigated. Incubation of prostate cancer cells with D-pinitol led to a significant decrease in the phosphorylation of FAK (Figure 4A,B). In addition, c-Src kinase activity was abolished by D-pinitol treatment in a dose-dependent manner (Figure 4C,D). Therefore, D-pinitol seems to act through a signaling pathway involving FAK and c-Src to inhibit cell migration of human prostate cancer cells.

### 2.5. NF-κB Is Involved in D-pinitol-Reduced Cell Migration in Prostate Cancers

**Figure 5:**
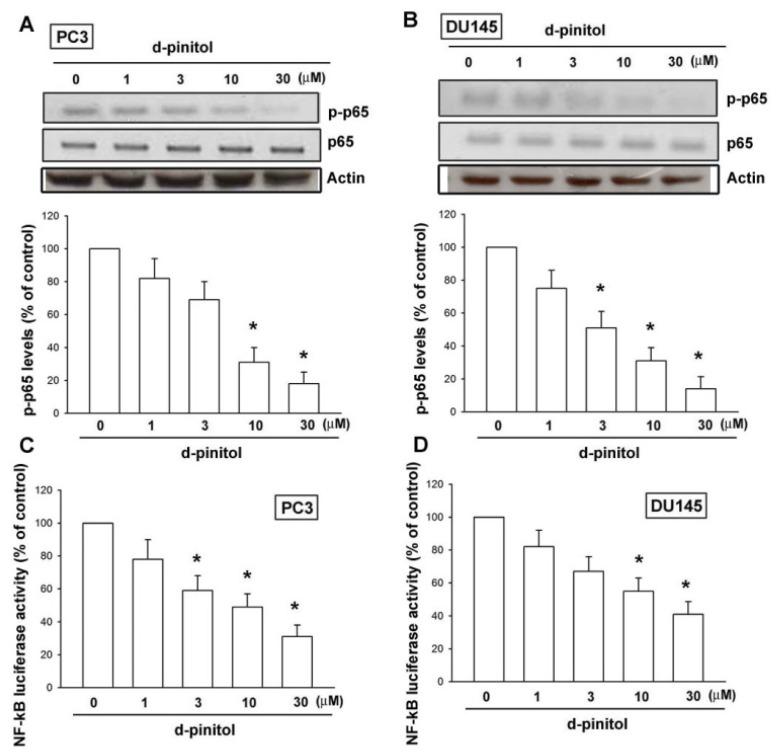
NF-κB mediates the response of human prostate cancer cells to D-pinitol. (**A** and **B**) Cells were incubated with various concentrations of D-pinitol for 24 h; the p-p65 expression was examined by Western blotting (*n* = 5). (**C** and **D**) Cells were incubated with various concentrations of D-pinitol for 24 h; the NF-κB activity was examined by NF-κB luciferase activity assay (*n* = 4). Results are expressed as the mean ± SE. *p < 0.05 compared with control.

D-pinitol has been reported to reduce lung cancer metastasis through the inhibition of NF-κB [19]. Therefore, the role of D-pinitol in NF-κB activation in prostate cancers was examined. Treatment of prostate cancer cells with D-pinitol reduced the phosphorylation of p-p65 (Figure 5A,B). NF-κB activation was further evaluated by analyzing NF-κB luciferase activity. Cells were transiently transfected with NF-κB-luciferase as an indicator of NF-κB activation. We observed that D-pinitol abolished NF-κB-luciferase activity (Figure 5C,D). Therefore, NF-κB is involved in D-pinitol-reduced metastasis in prostate cancer cells.

## 3. Discussion

Prostate cancer cells have a striking tendency to metastasize [1]. The development of novel therapeutic agents targeting the cell motility of prostate cancer cells is important for diminishing tumor metastasis. D-pinitol has various biological activities, such as anti-hyperlipidemic, antioxidant, cardioprotective and anti-inflammatory functions [14–16]. A recent study has shown that D-pinitol reduced metastasis of human lung cancer cells [19]. However, the anti-metastasis effects of D-pinitol on prostate cancer cells are mostly unknown. We found that D-pinitol inhibited cell migration and invasion at noncytotoxic concentrations (0 μM to 30 μM) in human prostate cancer cells. We also demonstrated that the downregulation of αvβ3 integrin, through the FAK, c-Src and NF-κB pathways, is involved in D-pinitol-reduced cancer migration. D-pinitol possesses activities that target different molecules in many cancer systems. Importantly, the current study is the first demonstration that the anti-metastasis activity of pinitol is mediated through the modulation of integrin. Whether the same effect of pinitol applies to other cancer systems needs further examination; however, our results provide a better understanding of the effects of pinitol. The results from this study indicate D-pinitol is a potential lead base on anti-metastasis activity in human prostate cancer cells with desirable pharmacological properties.

Integrins, which link the extracellular matrix to intracellular signaling molecules, regulate a number of cellular processes, including adhesion, signaling, motility, survival, gene expression, growth and differentiation [23,24]. It has been reported that disintegrin or a αvβ3 integrin antibody blocked cancer migration and metastasis [22,25]. In this study, we found that D-pinitol reduced the mRNA expression of αv and β3 integrin. Furthermore, treatment of prostate cancer cells with D-pinitol also diminished the cell surface expression of αvβ3 integrin. This indicates that αvβ3 integrin plays an important role in the D-pinitol-inhibited metastasis of prostate cancer cells.

Prostate cancer is prevalent in developed countries worldwide. Most prostate tumors remain confined to the prostate gland and adjacent soft tissue, causing little to no harm. However, nearly one in eight cases lead to metastasis, typically to bone [26]. The role of androgenic hormones in prostate cancer progression and survival has been reported previously and is supported by the ability of androgen ablation therapy to cause regression of both primary and metastatic disease [27]. In this study, we provide evidence that D-pinitol reduced the migration of two androgen-independent (PC3 and DU145) prostate cancer cells. However, we did not examine the role of D-pinitol in androgen-dependent prostate cancer cells. Whether D-pinitol reduces migration in androgen-dependent prostate cancer cells needs further examination.

FAK, a potential candidate signaling molecule, has been shown to be capable of regulating integrin-mediated signaling [28]. In addition, FAK-dependent c-Src activation is involved in tumor migration and invasion [21,22]. In the current study, we found that D-pinitol reduced FAK phosphorylation in a dose-dependent manner. Furthermore, D-pinitol also inhibited c-Src kinase activity in human prostate cancer cells. Taken together, our results provide evidence that D-pinitol downregulates cell motility and αvβ3 integrin expression in human prostate cancer cells via the FAK/c-Src signaling pathway.

A variety of growth factors stimulate cancer metastasis via signal-transduction pathways that converge to activate the NF-κB complex of transcription factors [29]. The results of this study show that NF-κB activation contributes to D-pinitol-inhibited migration of human prostate cancer cells. p65 is phosphorylated at Ser^536^ by a variety of kinases through various signaling pathways, and this enhances the p65 transactivation potential. The results of this study indicated that D-pinitol diminished the phosphorylation of p65. Furthermore, using transient transfection with NF-κB-luciferase as an indicator of NF-κB activity, we also established that D-pinitol-reduced NF-κB luciferase activity. Our data indicated that NF-κB activation might play an important role in cell migration and metastasis of human prostate cancer cells.

## 4. Experimental Section

### 4.1. Materials

Protein A/G beads, rabbit polyclonal antibodies specific for p-FAK, FAK, p-p65 and p65, were purchased from Santa Cruz Biotechnology (Santa Cruz, CA, USA). The pSV-β-galactosidase vector and luciferase assay kit were purchased from Promega (Madison, MA, USA). All other chemicals were purchased from Sigma-Aldrich (St. Louis, MO, USA).

### 4.2. Cell Culture

Human prostate cancer cell lines (PC3 and DU145) were purchased from the American Type Culture Collection. Cells were maintained at 37 °C, 5% CO_2_, in RPMI-1640 medium supplemented with 20 mM HEPES, 10% heat-inactivated fetal calf serum, 2 mM glutamine, 100 U/mL penicillin and 100 μg/mL streptomycin.

### 4.3. MTT Assay

Cell viability was determined with the 3-(4,5-dimethylthiazol-2-yl)-2,5-diphenyltetrazolium bromide (MTT) assay [30,31]. After treating with D-pinitol for 24 or 48 h, cultures were washed with PBS. Then, MTT (0.5 mg/mL) were added to each well, and the mixture was incubated at 37 °C for 2 h. To dissolve formazan crystals, culture medium was then replaced with an equal volume of DMSO. After the mixture was shaken at room temperature for 10 min, the absorbance of each well was determined at 550 nm using a microplate reader (Bio-Tek, Winooski, VT, USA).

### 4.4. TUNEL Assay

The terminal deoxynucleotidyl transferase-mediated deoxyuridine triphosphate nick-end labeling (TUNEL) assay was used to examine the cell apoptosis by using the BD ApoAlert™ DNA Fragmentation Assay Kit. Briefly, cells were incubated with D-pinitol for 24 h. The cells were trypsinized, fixed with 4% paraformaldehyde and permeabilized with 0.1% Triton-X-100 in 0.1% sodium citrate. After being washed, the cells were incubated with the reaction mixture for 60 min at 37 °C. The stained cells were then analyzed with flow cytometer.

### 4.5. Caspase 3 Activity Assay

The assay is based on the ability of active enzyme to cleave chromophore from enzyme substrate, Ac-DEVD-pNA. Cell lysates were prepared and incubated with anti-caspase 3. Immunocomplexes were incubated with peptide substrate in assay buffer (100 mM NaCl, 50 mM 4-(2-hydroxyethyl)-1-piperazine-ethanesulfonic acid [HEPES], 10 mM dithiothreitol, 1 mM EDTA, 10% glycerol, 0.1% 3-[32]-1-propanesulfonate [CHAPS], pH 7.4) for 2 h at 37 °C. The release of *p*-nitroaniline was monitored at 405 nm. The results are the percent change in activity compared to the untreated control.

### 4.6. Migration and Invasion Assay

The migration assay was performed using Transwell inserts (Costar, NY, USA; 8-mm pore size) in 24-well dishes. For invasion assay, filters were precoated with 30 μL Matrigel basement membrane matrix (BD Biosciences, Bedford, MA, USA) for 30 min. The following procedures were the same for both migration and invasion assays. After the treatment with D-pinitol (0, 1, 3, 10 and 30 μM) for 24 h, cells were harvested and seeded to Transwell at 1 × 10^4^ cells/well in serum-free medium and then incubated for 24 h at 37 °C in 5% CO_2_. Cells were then fixed in 3.7% formaldehyde for 5 min and stained with 0.05% crystal violet in PBS for 15 min. Cells on the upper side of the filters were removed with cotton-tipped swabs, and the filters were washed with PBS. Cells on the underside of the filters were examined and counted under a microscope. Each experiment was performed in triplicate and repeated at least three times.

### 4.7. Wound-Healing Migration Assay

For the wound-healing migration assay, cells were seeded on 12-well plates at a density of 1 × 10^5^ cells/well in culture medium. At 24 h after seeding, the confluent monolayer of culture was scratched with a fine pipette tip, and migration was visualized by microscope and magnification. The rate of wound closure was observed at the indicated time.

### 4.8. Flow Cytometric Analysis

Human prostate cells were grown in 6-well dishes and then washed with PBS and detached using trypsin at 37 °C. Cells were fixed for 10 min in PBS containing 3.7% paraformaldehyde, rinsed in PBS and incubated with mouse anti-human αvβ3 integrin (1:100) (BD Biosciences, San Jose, CA, USA) for 1 h at 4 °C. Cells were then washed in PBS and incubated with fluorescein isothiocyanate-conjugated goat anti-mouse secondary IgG (1:100; Leinco Technologies, St. Louis, MO, USA) for 45 min at 4 °C. After a final rinse, cells were analyzed using a FACSCalibur flow cytometer and CellQuest software (BD Biosciences, San Jose, CA, USA).

### 4.9. Western Blot Analysis

Cellular lysates were prepared and proteins were resolved by SDS-PAGE [32,33]. Proteins were transferred to Immobilon polyvinylidene fluoride membranes. The blots were blocked with 4% bovine serum albumin for 1 h at room temperature and probed with rabbit anti-human antibodies against p-FAK, FAK, p-p65 or p65 (1:1000) for 1 h at room temperature (Santa Cruz, CA, USA). After three washes, the blots were incubated with peroxidase-conjugated donkey anti-rabbit secondary antibody (1:1000) for 1 h at room temperature. The blots were visualized with enhanced chemiluminescence using X-OMAT LS film (Eastman Kodak, Rochester, NY, USA). Quantitative data were obtained using a computing densitometer and ImageQuant software (Molecular Dynamics, Sunnyvale, CA, USA).

### 4.10. Kinase Activity Assay

c-Src activity was assessed with a c-Src Kinase Activity Assay kit (Abnova, Taipei, Taiwan). The kinase activity kits are based on a solid phase ELISA that uses a specific synthetic peptide as a substrate for c-Src and a polyclonal antibody that recognizes the phosphorylated form of the substrate.

### 4.11. Quantitative Real-Time PCR

Total RNA was extracted from prostate cancer cells using a TRIzol kit (MDBio, Taipei, Taiwan). Reverse transcription was performed using 1 μg total RNA and an oligo(dT) primer [20,31]. Quantitative real-time PCR (qPCR) was carried out using a TaqMan One-step PCR Master Mix (Applied Biosystems, Foster City, CA, USA). Total cDNA (100 ng) was added to each 25 μL reaction with sequence-specific primers and TaqMan probes. All target gene primers and probes were purchased commercially, including those for (GAPDH as an internal control (Applied Biosystems). qPCR was carried out in triplicate with a StepOnePlus (Applied Biosystems) sequence detection system. The cycling conditions were 10 min at 95 °C, followed by 40 cycles of 95 °C for 15 s and 60 °C for 60 s. To calculate the cycle number at which the transcript was detected (*C*_T_), the threshold was set above the non-template control background and within the linear phase of target gene amplification.

### 4.12. Reporter Gene Assay

The prostate cancer cells were transfected with NF-κB reporter plasmid using Lipofectamine 2000 according to the manufacturer’s recommendations. Twenty-Four hours after transfection, the cells were treated with inhibitors for 30 min, and then D-pinitol or vehicle was added for 24 h. Cell extracts were then prepared and luciferase, and β-galactosidase activities were measured.

### 4.13. Statistical Analysis

Data are presented as the mean ± the standard error of the mean (SEM). Statistical analysis between two samples was performed using the Student’s *t*-test. Statistical comparisons of more than two groups were performed using one-way analysis of variance with Bonferroni’s post-hoc test. In all cases, *p* < 0.05 was considered significant.

## 5. Conclusions

Natural product drugs have been suggested to play a dominant role in pharmaceutical care [34]. Natural products are an important source of potential cancer chemotherapeutic and cancer metastasis agents. The present study demonstrated that D-pinitol inhibits the migration and invasion of human prostate cancer cells. The downregulation of αvβ3 integrin through the FAK, c-Src and NF-κB pathways are involved in D-pinitol-mediated effects. Therefore, D-pinitol may show beneficial effects in reducing the metastatic activity of human prostate cancer cells.
